# Raman Mapping-Based Reverse Engineering Facilitates Development of Sustained-Release Nifedipine Tablet

**DOI:** 10.3390/pharmaceutics14051052

**Published:** 2022-05-13

**Authors:** Ningyun Sun, Liang Chang, Yi Lu, Wei Wu

**Affiliations:** 1 Key Laboratory of Smart Drug Delivery of MOE, School of Pharmacy, Fudan University, Shanghai 201203, China; sunningyun@sphsine.com; 2 SPH Sine Pharmaceutical Laboratories Co., Ltd., Shanghai 201206, China; changliang@sphsine.com; 3 Fudan Zhangjiang Institute, Shanghai 201203, China

**Keywords:** Raman mapping, reverse engineering, particle size, sustained release, bioequivalence

## Abstract

The development of generic preparations that are bioequivalent to a reference listed drug (RLD) is faced with challenges because some critical attributes of RLDs are commonly unknown to developers. In order to determine these attributes, Raman mapping-based reverse engineering in this study to analyze a model sustained-release tablet of nifedipine. The Raman mapping results indicate that the size and size distribution of nifedipine are critical to its release pattern and bioavailability. The tablets with a particle size of nifedipine comparable to that of a commercial product, Adalat^®^-L, showed similar in vitro release profiles to the RLD. Moreover, a pharmacokinetic study in human volunteers proved the bioequivalence of the two preparations. In conclusion, Raman mapping-based reverse engineering has the potential to facilitate the development of generic preparations.

## 1. Introduction

Nowadays, generic drugs hold the leading position in clinics due to their therapeutic equivalence and huge economic benefits [1,2]. In the year 2014, USD 254 billion was saved because of the use of generic drugs in the United States [3]. However, achieving pharmaceutical equivalence and bioequivalence to reference listed drugs (RLDs) remains challenging [4,5]. Thus far, dissolution testing is one of the dominant screening methods during the development of generic drugs. Nevertheless, establishing a workable in vitro dissolution method that meets the requirements of in vitro–in vivo correlation (IVIVC) is difficult. The overall qualification rate of IVIVC submitted to the Food and Drug Administration (FDA) of the U.S. is only 40% [6]. A trial-and-error method thus prevails in the development of generic drugs, leading to huge financial and time costs. The goal of the development of a generic drug product is comparative consistency with the RLD to meet the quality target profile. Identification of the critical quality attributes (CQAs) of RLDs is crucial for the development of generic drugs [7].

Acquiring the CQAs of RLDs is difficult. The package insert, patent files, and literature provide useful information to establish the initial formula. CQAs, such as particle size and size distribution of active pharmaceutical ingredients (APIs), crystal forms, and spatial distribution of APIs in the matrix of excipient, are unavailable, but are critical for the development of oral solid-dosage forms, particularly for poorly soluble APIs. For example, the particle size [8,9] and crystal form [10,11] strongly impact the dissolution [12,13] and, consequently, the oral bioavailability of APIs [14]. The spatial distribution of APIs in the excipient matrix is of good reference value for process development [15,16]. In this instance, reverse engineering of an RLD may facilitate the development of generic drugs by providing essential critical product attributes.

Raman scattering is the inelastic scattering of the monochromatic light, which is a shift in the energy of the incident radiation as a result of interaction with vibrations in the molecule. Raman mapping works by recording point-by-point spectra from different areas of a sample mounted upon a moveable stage. The obtained spectra are converted to images through various mathematical approaches [17]. The point-by-point mapping approach is the most commonly used technique when employing Raman mapping [18]. Raman spectroscopy and mapping are valuable tools in the pharmaceutical field [19,20], such as non-destructive analysis of chemical composition and molecular structure [21,22,23,24,25,26], quantitative analysis of critical attributes [27,28,29,30,31,32,33], identification of polymorphs or in situ monitoring of crystallization [34,35,36], real-time release testing [37,38], and real-time process monitoring and control [39,40,41,42,43]. Therefore, Raman mapping can be used as a powerful tool in reverse engineering of an RLD to provide valuable information on CQAs and processing parameters.

Adalat^®^-L is a sustained-release tablet of nifedipine for the treatment of hypertension. Being the first generation of nifedipine preparation, the preparation technology of Adalat^®^-L is different from that of subsequent preparations, i.e., Adalat^®^-CC (a coat-core tablet) and Adalat^®^-LA (an osmotic pump tablet). No functional excipients are listed in the package insert of Adalat^®^-L. Preliminary tests indicated that the tablet rapidly disintegrates in the dissolution medium, which implies that the tablet is not a matrix-based sustained-release delivery system. Because nifedipine is poorly water-soluble, the sustained release is attributed to the particle size and size distribution, well-controlled to meet the sustained-release requirement, of the API nifedipine [44]. Particle size is a critical attribute in the development of generic preparation of nifedipine. Measuring the size of nifedipine in Adalat^®^-L is challenging due to the interference from other excipients. The development of generic nifedipine preparations is extremely difficult as the inconsistent size and size distribution of nifedipine inevitably lead to an alteration in drug release pattern and a lack of bioequivalence. Although a trial-and-error method may be applied in the process, its time and money costs are huge. Conversely, Raman mapping-based reverse engineering provides a good solution to the problem by providing valuable information on the size and size distribution, as well as the distribution pattern, of nifedipine in Adalat^®^-L.

In this study, we obtained Raman spectra of each component in Adalat^®^-L for Raman mapping. We reverse analyzed the CQAs, such as the particle size and distribution of nifedipine, and the content of each excipient in Adalat^®^-L, via Raman mapping. Based on the information, we prepared generic tablets with different particle sizes of nifedipine. We compared the release profiles of the prepared tablets with that of the RLD. Finally, we conducted a bioequivalence study to confirm the agreement between the optimal batch and the RLD.

## 2. Materials and Methods

### 2.1. Materials

We purchased Adalat^®^-L (20 mg) from Bayer AG, Osaka, Japan; nifedipine from Changzhou Siyao Pharmaceuticals Co., Ltd., Changzhou, China; microcrystalline cellulose from DuPont Nutrition USA, Inc., Newark, DE, USA; lactose from DMV-Fonterra Excipients GmbH & Co. KG, Noerten-Hardenberg, Germany; polysorbate 80 from Longyou Juxing Cereal & Oil Medicine Chemical Co., Ltd., Quzhou, China. We bought corn starch and magnesium stearate from Haiyan Liuhe Pharmaceutical Co., Ltd., Jiaxing, China; Opadry^®^ from Shanghai Coloron Coating Technology Co., Ltd., Shanghai, China. We performed all the preparations and analyses in the dark due to the photosensitivity of nifedipine.

### 2.2. Raman Mapping of Adalat^®^-L

We performed Raman mapping using a Laser Microscopic Confocal Raman Spectrometer (inVia, Renishaw, Gloucestershire, UK). We excited the Raman scattering with a 633 nm laser operated at 100% laser power and a 50× objective lens used to collect the backscattered light. We performed Raman point-by-point mapping with a step size of 4 μm in an area of 1000 × 1000 μm. The acquisition time per point was 0.5 s. We recorded scans in a spectral window from 603 to 1738 cm^−1^. We acquired and analyzed the data using WiRE 5.2 software, which was affiliated with the Raman spectrometer. We obtained the reference Raman spectra of nifedipine and the three most abundant excipients, corn starch, lactose, and microcrystalline cellulose, by measuring each pure sample using similar measurement conditions. We obtained the Raman image data of Adalat^®^-L by scanning the tablet after being cut using a scalpel. We calculated the particle sizes of each component in the tablet using the statistical tool in the test software. Additionally, we estimated the content of each component using the software according to the matching degree with the reference spectra.

### 2.3. Preparation of Nifedipine Sustained-Release Tablets

We produced four sets of tablets with different particle sizes of nifedipine to investigate its quality attributes. We pulverized the nifedipine using GF-300A high-efficiency universal crusher (Shanghai Tianhe Pharmaceutical Machinery Co., Ltd., Shanghai, China), which we sieved with different meshes (75, 125, 425, and 1000 μm). Then, we measured the particle size and size distribution of the sieved nifedipine using a Malvern FPIA-3000 (Malvern, UK). We collected and compared the particle size data (D90, D50, and D10).

We prepared the core tablets by wet granulation. Because the measured weight of the core tablet of Adalat^®^-L was 79.4 ± 0.6 mg, we set the weight of the core tablet to 80 mg. The composition determined based on the Raman mapping results is shown in Table 1. We mixed nifedipine with polysorbate 80, microcrystalline cellulose, lactose, and corn starch for several minutes. We added starch slurry (15%, *w*/*w*) to the mixture to prepare the soft material, which we sieved through 14 mesh to obtain wet granules. We dried the wet granules at 65 °C until the moisture content was less than 3.5% (*w*/*w*). Following the sieving through 14-mesh sieves, we mixed the dry granules with magnesium stearate. We compressed the mixture into tablets using a rotary tablet compression machine (Beijing Gylongli Sci. & Tech. Co., Ltd., Beijing, China). We adjusted the tablet press to produce tablets with 3.0–6.0 kp hardness. We then coated the core tablets with Opadry^®^ (Shanghai Coloron Coating Technology Co., Ltd., Shanghai, China) in a high-efficiency coating machine (Wenzhou Pharmaceutical Machinery Factory, Wenzhou, China). We set the coating weight gain to 3.0–4.0% (*w*/*w*).

### 2.4. Validation of the Prepared Tablets by Raman Mapping

We mapped the tablets using a Raman spectrometer to compare the particle size with that of Adalat^®^-L. We also studied the crystallinity of the nifedipine in preparation by extracting the spectrum of nifedipine from the tablets.

### 2.5. In Vitro Dissolution Studies

We determined dissolution by adapting the method recommended by the Ministry of Health and Welfare of Japan. We adopted 900 mL pH 4.0 acetate buffer with 0.05% polysorbate 80 as the release medium. We set the rotation rate of the paddle to 50 rpm. We removed 5 mL samples at predetermined intervals, while we supplemented an equal volume of blank media with the same temperature. We filtered the samples for measurement of nifedipine concentration by HPLC (Agilent Infinity 1260 series, Agilent Technologies, Santa Clara, CA, USA) at 235 nm. We used a reverse-phase C18 column (4.6 × 150 mm, 5 μm) for separation. The mobile phase consisted of a mixture of methanol and water (60:40, *v*/*v*), which was pumped at a flow rate of 1.0 mL/min. The standard curve for nifedipine was linear over the concentration range of 2.8~28.0 μg/mL, and the correlation coefficient was higher than 0.999. We obtained the accuracy and precision by measuring three different concentrations of nifedipine (2.8, 11.2, and 28 μg/mL), which ranged from 100.3% to 100.8% and 1.1% to 1.3%, respectively.

We used the similarity factor (*f*_2_) to test the similarity between two dissolution profiles [45].

(1) $$f_{2}=50log\left\{{\left[1+\frac{1}{n}\sum_{t=1}^{n}{\left(R_{t}-T_{t}\right)}^{2}\right]}^{-0.5}\times 100\right\}$$

where *R_t_* and *T_t_* represent the dissolution of reference and test preparation at different time points, respectively; *n* is the number of observations. An *f*_2_ value higher than 50 indicates similarity between two dissolution curves. For the calculation of *f*_2_ values, we requested at least 12 individual dosage units, and we considered only one data point after 85% of the drug was released.

### 2.6. Bioequivalence Studies

We conducted an open-label, randomized, two-period, two-sequence, single-dose, two-way, crossover comparative bioequivalence study on healthy human volunteers. We obtained ethical approval for this study from the affiliated hospital of Xuzhou Medical University (Protocol approval No. XYFY2018-YL077-01 for fasting study and XYFY2018-YL078-01 for fed study). We compared the pharmacokinetic data of the prepared tablet with those of Adalat^®^-L. Twenty-eight volunteers enrolled in the fasting study, and another twenty-eight in the fed study. We obtained written informed consent from each volunteer after explaining the objectives of the study. We medically screened all volunteers to establish their fitness for the study.

We randomly divided the twenty-eight volunteers into two groups. One group received Adalat^®^-L, while the other received the prepared tablet during the first treatment period under fasting/fed conditions. After a washout period of 7 days, the volunteers exchanged formulations during the second treatment period. We withdrew blood samples at 0.5, 1, 1.5, 2, 2.5, 3, 3.5, 4, 4.5, 5, 6, 8, 10, 12, 14, 16, 24, and 36 h after drug administration into vacutainer tubes containing anticoagulant. We immediately centrifuged the blood samples at 1900× *g* for 10 min at 4 °C. We froze the plasma at −20 °C pending content analysis via LC-MS/MS.

To extract nifedipine from the biosamples, we added a 100 μL aliquot of human plasma to a deep well plate containing 5 μL internal standard (0.2 ng/μL of nifedipine-d6). Then, we added 500 μL acetonitrile to the plasma, which vortexed for about 1 min. We centrifuged the mixture (20 °C) at 3000 rpm for 10 min. We diluted a 100 μL aliquot of the supernatant with 500 μL acetonitrile/H_2_O/formic acid (45/55/0.2, *v*/*v*), which vortexed for 1 min. We centrifuged the mixture (20 °C) at 3000 rpm for 5 min, and we estimated the drug concentration in the supernatant by LC-MS/MS method.

We used an Exion LC^TM^ system (AB SCIEX, Framingham, MA, USA), coupled with a TRIPLE QUAD^TM^ 6500 + mass spectrometer (AB SCIEX, Framingham, MA, USA), with IonDrive^TM^ Turbo V^TM^ ion source, for the LC-MS/MS analysis. We conducted chromatographic separation at 40 °C with a gradient mobile phase on an Agilent ZOBAX XDB-C18 column (column size: 2.1 × 50 mm). The mobile phase (A) consisted of H_2_O/formic acid at a volume ratio of 100/0.2, whereas (B) consisted of acetonitrile/formic acid at a volume ratio of 100/0.2. The flow rate was 0.4 mL/min, and we programmed the mobile phase to linearity changes as follows: 55% (A) at 0–0.9 min, 55–10% (A) at 0.9–1 min, 10% (A) at 1–1.5 min, 10–55% (A) at 1.5–1.6 min, and 55% (A) at 1.6–3.5 min. The injection volume was 10 μL. We used Analyst version 1.6.3 for data acquisition and analysis.

We subjected the plasma drug concentration–time data to non-compartmental analysis using pharmacokinetic software WinNonin^®^ version 6.4 to obtain various pharmacokinetic parameters.

We conducted statistical analysis using SAS^®^ software version 9.4 (SAS Institute Inc., Cary, NC, USA). We used the general linear model to analyze the pharmacokinetic parameters such as AUC_0–t,_ AUC_0–∞_, and C_max_ after natural logarithm transformation. As for these parameters, we considered results with a 90% confidence interval within the scope of 80.00~125.00% to be bioequivalent to Adalat^®^-L.

## 3. Results and Discussion

### 3.1. Analysis of Adalat^®^-L by Laser Raman Spectroscopy

**Figure 1:**
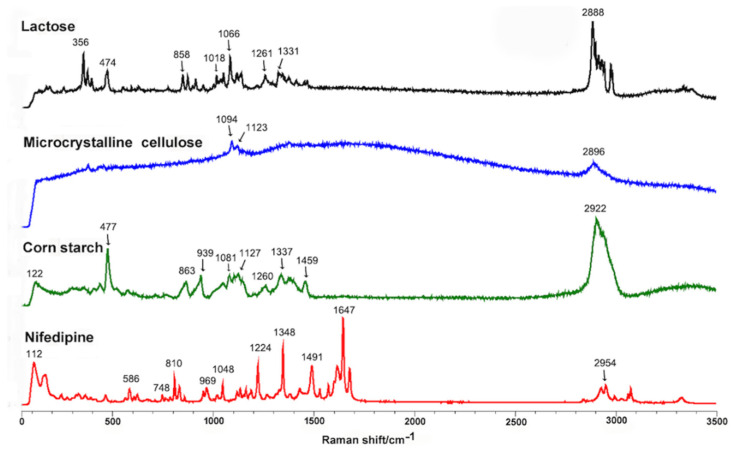
Raman spectra of nifedipine, corn starch, microcrystalline cellulose, and lactose. The characteristic bands are marked: nifedipine, 112, 586, 748, 810, 969, 1048, 1224, 1348, 1491, 1647, and 2954 cm^−1^; corn starch, 122, 477, 863, 939, 1081, 1127, 1260, 1337, 1459, and 2922 cm^−1^; microcrystalline cellulose, 1094, 1123, and 2896 cm^−1^; lactose, 356, 474, 858, 1018, 1066, 1261, 1331, and 2888 cm^−1^.

**Figure 2:**
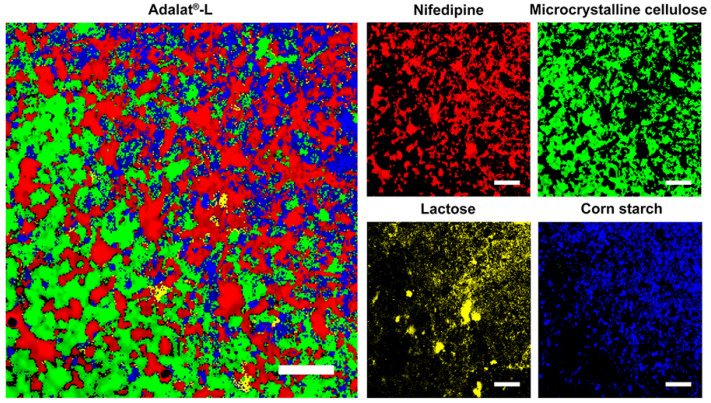
Raman mapping of Adalat^®^-L, and distributions of nifedipine, microcrystalline cellulose, lactose, and corn starch. Scale bar is 200 µm.

Prior to Raman-mapping experiments, we optimized the instrumental conditions for pure nifedipine and the main excipients in all spectroscopic analyses. For this purpose, we performed Raman spectroscopy on nifedipine and excipients in powder form in order to monitor the components that were present in the tablets. Figure 1 shows the specific Raman spectrum of pure nifedipine, corn starch, microcrystalline cellulose, and lactose. We used direct classic least squares method (DCLS) to produce the Raman images from over 63,001 collected spectra. The characteristic bands for nifedipine, corn starch, microcrystalline cellulose, and lactose are marked in Figure 1. Because the bands mainly located between 603 and 1738 cm^−1^, we adopted this region for Raman mapping. Figure 2 shows the scanning distribution of each component of Adalat^®^-L. The corresponding materials with different colors are shown as follows: red, nifedipine; blue, corn starch; yellow, lactose; and green, microcrystalline cellulose. We statistically analyzed the particle size by imaging analysis of the chemometrics data, and estimated the content of each component by Raman chemical imaging analysis. The particle size and estimated content of nifedipine and the three main obtained excipients are shown in Table 2 and Table 3. According to the Raman analysis, the particle size (D90) of nifedipine in Adalat^®^-L was 150 μm, and the estimated contents of nifedipine, microcrystalline cellulose, lactose, and corn starch were 33.94%, 33.02%, 1.40%, and 31.65%, respectively.

### 3.2. Comminution and Particle Size Control of Nifedipine

According to the measured size in Adalat^®^-L, we prepared nifedipine with different particle sizes (Table 4). We set the maximum size to a D (90) close to 150 μm. Tablets with different sizes of nifedipine helped us to investigate the effect of particle size on the Raman spectroscopy, and characteristics of in vitro release and in vivo absorption.

### 3.3. Validation by Raman Mapping of Prepared Tablets

**Figure 3:**
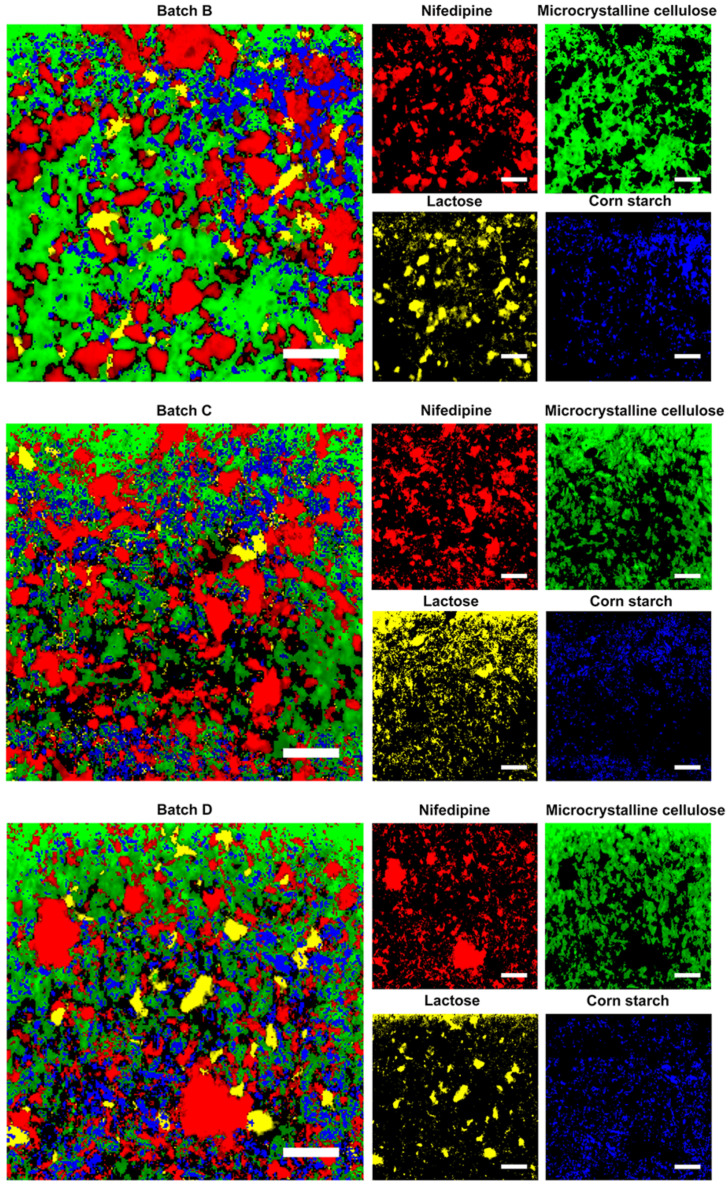
Raman mapping of the generic preparations, and distributions of nifedipine, microcrystalline cellulose, lactose, and corn starch. Scale bar is 200 µm.

We prepared four batches of sustained-release tablet with different sizes of nifedipine. First, we disintegrated and dispersed the tablets in water. We measured the size of the dispersed nifedipine. We visual observed that the suspension obtained from the batch A tablet was more turbid than that from other preparations. Thus, we did not map Batch A by the Raman spectrometer, but was further evaluated in dissolution. Figure 3 shows the scanning of the tablets and distribution of each component in the prepared tablets. The obtained particle size of nifedipine is shown in Table 5.

We observed the different particle sizes of nifedipine among the prepared tablets from the D (90) data. The trend was consistent with that of the size of the raw materials measured by a Malvern FPIA-3000S. The result revealed differences between the particle size obtained from Raman spectroscopy and that from the Malvern instrument. This could be partly due to the difference in the detecting principle, and to the change in particle sizes under physical stress during formulation processes such as mixing, granulation, drying, blending, and tableting. The results also showed that the particle size of Batch C was closest to that of Adalat^®^-L. The particle sizes of Batches B and D were significantly larger or smaller than that of Adalat^®^-L. We estimated that the release behavior of Batch C should be similar to that of Adalat^®^-L, whereas the release behaviors of Batches B and Batch D should be slower or faster than that of Adalat^®^-L, respectively.

**Figure 4:**
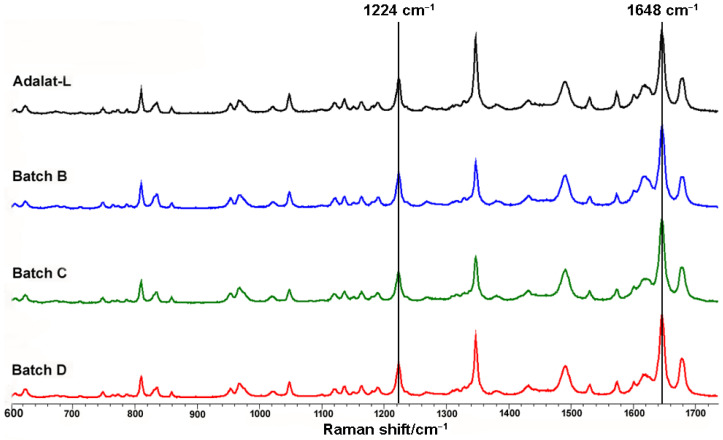
Comparison of crystalline forms of sustained-release nifedipine tablets.

The crystalline forms of nifedipine in different tablets are also compared in Figure 4. We extracted the Raman spectra of nifedipine from each batch of sustained-release nifedipine tablets. The peaks of each spectrum were basically the same, indicating the consistent solid form. Additionally, the C–C–O stretch at 1224 cm^−1^ and the C=C stretch at 1648 cm^−1^ both comply with the characteristics of the α-form of nifedipine crystal [46].

### 3.4. In Vitro Dissolution Studies

**Figure 5:**
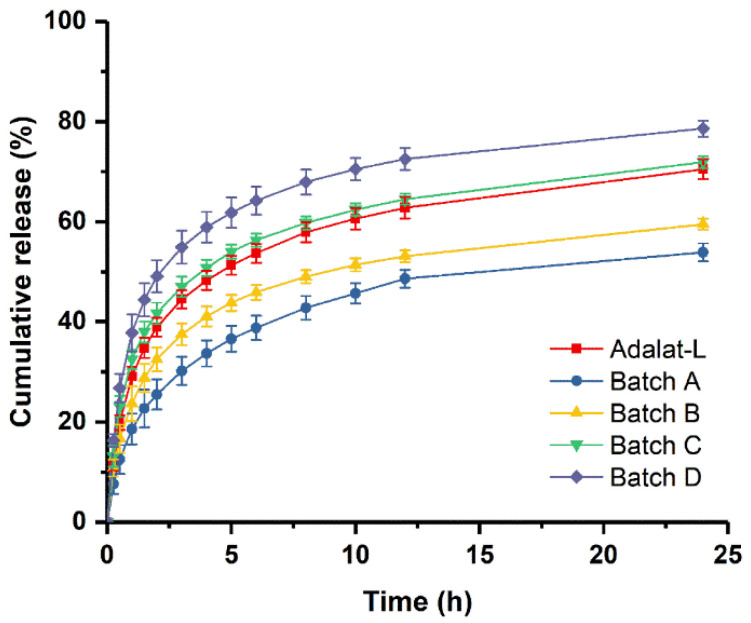
Comparison of in vitro dissolution profiles between prepared tablets and Adalat^®^-L (*n* = 12).

The release profiles of the prepared tablets are compared with that of Adalat^®^-L in Figure 5. The release profile of each preparation (the similarity factor *f*_2_ was 54.9, 78.2, and 52.3 for Batches B, C, and D, respectively) was similar to that of Adalat^®^-L, but not that of Batch A (the similarity factor *f*_2_ of Batch A was 44.2). The release behavior of Batch C was the most consistent. Compared with Adalat^®^-L, Batches B and D showed significant differences in the absolute value of cumulative release at each time point, particularly after 1 h of release. Taking 4 and 8 h as examples, the cumulative release of Batch B was about 7.2% and 8.9% lower than that of Adalat^®^-L, respectively. The cumulative release of Batch D was about 10.6% and 10.0% higher than that of Adalat^®^-L, respectively. Because nifedipine is a BCS Class II drug, there is a certain correlation between the drug release rate and the absorption rate in vivo. Therefore, although the similarity factor *f*_2_ of each release profile (Batches B, C, and D) was greater than 50, the difference in the absolute value of release at each time point might cause a huge difference in oral bioavailability.

### 3.5. Bioequivalence Studies

**Figure 6:**
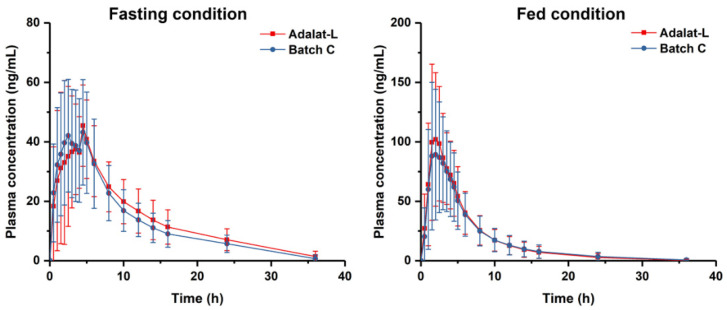
Plasma concentration-time profile under fasting ad fed conditions.

According to the results of the in vitro studies, such as dissolution studies and Raman spectroscopy, we chose the prepared tablets from Batch C to conduct the bioequivalence studies with Adalat^®^-L. The plasma concentration versus time profiles of Batch C and Adalat^®^-L were similar and nearly superimposable (Figure 6). The pharmacokinetic parameters of these formulations are presented in Table 6.

Under the fasting condition, the peak plasma concentration (C_max_) after oral single-dose administration of the two formulations (Batch C and Adalat^®^-L) in 28 healthy human volunteers was 56.4 ± 15.4 ng/mL and 54.9 ± 16.3 ng/mL, respectively. There was no significant difference in the C_max_ of the two formulations under fasting conditions. The area under the curve (AUC_0–t_) was 443.4 ± 150.0 and 478.1 ± 156.2 ng/mL·h, respectively; the AUC_0–∞_ was = 501.6 ± 147.8 and 536.6 ± 162.8 ng/mL·h, respectively.

Under the fed condition, the C_max_ was 107.5 ± 46.5 and 119.8 ± 44.1 ng/mL for Batch C and Adalat^®^-L respectively. There was also no significant difference in the peak plasma of the two formulations under the fed condition. The AUC_0–t_ was 539.6 ± 303.4 and 569.3 ± 283.0 ng/mL·h, respectively; the AUC_0–∞_ was 569.1 ± 314.9 and 597.3 ± 297.3 ng/mL·h, respectively.

Thus, from the results, we concluded that the C_max_, AUC_0–t_, and AUC_0–∞_ of Batch C were within the 90% confidence interval of bioequivalence criteria compared with Adalat^®^-L. The prepared tablet Batch C was bioequivalent with Adalat^®^-L both under fasting and fed conditions, which was consistent with the findings of the in vitro study.

## 4. Conclusions

In this study, we revealed the critical attributes of Adalat^®^-L, such as the size distribution of nifedipine and the contents of main excipients, by Raman-mapping-based reverse engineering. Although the particle size of nifedipine in the optimal batch was not consistent with that of the RLD obtained by Raman images, the information was advantageous for determining the initial formula of the generic preparation. We prepared four sets of tablets with different particle sizes of nifedipine for screening, starting from the obtained RLD result. The tablet that was closest in nifedipine size to Adalat^®^-L showed a similar release pattern to the RLD. We also achieved bioequivalence between the generic preparation and the RLD in both fasting and fed conditions. In conclusion, Raman mapping may primarily facilitate the development of generic preparation in two ways: reverse analysis of the critical attributes of RLDs and confirmation of consistency between generic drugs and RLDs. This study was an exploration, providing experience for the better use of Raman mapping in future reverse engineering.

## Figures and Tables

**Table 1:** Composition of core tablets.

Ingredients	Amount (mg)
Nifedipine	20.0
Polysorbate 80	0.4
Microcrystalline cellulose	26.0
Lactose	8.0
Corn starch (for blending)	12.4
Corn starch (for starch slurry)	12.4
Magnesium stearate	0.8
SUM	80.0

**Table 2:** Particle size of nifedipine in Adalat^®^-L.

	D90 (μm)	D50 (μm)	D10 (μm)
Nifedipine	150	118	30.7

**Table 3:** Estimated content of each component in Adalat^®^-L.

Components	Nifedipine	Microcrystalline Cellulose	Lactose	Corn Starch
Proportion (%)	33.94	33.02	1.40	31.65

**Table 4:** Particle size of raw nifedipine for preparation of each batch.

Batch	Particle Size		
	D (90) (μm)	D (50) (μm)	D (10) (μm)
A	145.2 ± 0.6	69.3 ± 0.6	12.1 ± 0.5
B	96.1 ± 0.7	38.2 ± 0.6	8.4 ± 0.6
C	62.0 ± 0.6	26.3 ± 0.2	5.5 ± 0.3
D	38.9 ± 0.5	18.8 ± 0.4	6.1 ± 0.4

**Table 5:** Particle size of nifedipine in prepared tablets and Adalat^®^-L.

	Particle Size		
	D (90) (μm)	D (50) (μm)	D (10) (μm)
Adalat^®^-L	150	118	30.7
Batch B	173	118	52.2
Batch C	147	121	47.5
Batch D	108	103	32.7

**Table 6:** Pharmacokinetics parameters analysis of prepared Batch C and Adalat^®^-L tablets under fasting and fed conditions.

Condition	Pharmacokinetic Parameters	Mean and Ratio			90% Confidence Interval
		Batch C (T)	Adalat^®^-L (R)	(T/R)%	
Fasting (N = 28)	C_max_ (ng/mL)	56.4 ± 15.4	54.9 ± 16.3	102.75	92.15~114.56
	AUC_0–t_ (ng/mL·h)	443.4 ± 150.0	478.1 ± 156.2	92.73	86.67~99.21
	AUC_0–∞_ (ng/mL·h)	501.6 ± 147.8	536.6 ± 162.8	93.48	87.55~99.80
Fed (N = 28)	C_max_ (ng/mL)	107.5 ± 46.5	119.8 ± 44.1	89.67	81.95~98.11
	AUC_0–t_ (ng/mL·h)	539.6 ± 303.4	569.3 ± 283.0	94.78	88.39~101.63
	AUC_0–∞_ (ng/mL·h)	569.1 ± 314.9	597.3 ± 297.3	95.28	88.97~102.04

## Data Availability

The data presented in this study are available within this article. Any additional requests for data will receive a prompt response from the corresponding author.
